# Effects of bolus injection rate on the pharmacodynamics and pharmacokinetics of propofol, etomidate and sufentanil in rats

**DOI:** 10.3389/fvets.2025.1696260

**Published:** 2025-12-08

**Authors:** Sicheng Liu, Deying Gong, Xiaoxiao Li, Feng Qiu, Wensheng Zhang

**Affiliations:** 1 Department of Anesthesiology, West China Hospital, Sichuan University, Chengdu, China,; 2 Laboratory of Anesthesia and Critical Care Medicine, National-Local Joint Engineering Research, Centre of Translational Medicine of Anesthesiology, West China Hospital, Sichuan University, Chengdu, China

**Keywords:** innovative drug research and development, injection rate, anesthesia, propofol, etomidate, sufentanil, rat

## Abstract

**Introduction:**

The preclinical phase of innovative drug research and development involves a comprehensive assessment of multiple variables that may influence therapeutic outcomes and safety profiles. Although injection rate represents a potentially modifiable parameter in pharmacological studies, its specific effects on experimental outcomes remain insufficiently characterized in animal models. This study systematically explored the relationship between the intravenous injection rates of three anesthetics and their pharmacodynamic and pharmacokinetic responses in rats.

**Methods:**

Three anesthetics were administered to rats via intravenous bolus at varying rates: fast (0.06 mL/s), medium (0.02 mL/s), and slow (0.01 mL/s). Quantitative behavioral assessments were conducted to determine onset latency and duration of anesthesia. Comprehensive safety evaluations included invasive hemodynamic monitoring, respiratory frequency measurements, and myoclonus scoring. Pharmacokinetic profiling was performed using plasma samples analyzed by validated HPLC and HPLC-MS techniques.

**Results:**

Faster injection significantly altered pharmacodynamic profiles, with the fast group showing shorter onset latency and longer duration of effect compared to the slow group. However, this kinetic advantage was associated with a higher incidence of adverse events, including transient hypotension, increased respiratory depression, and more severe myoclonus. Pharmacokinetic analyses revealed dose-rate-dependent plasma concentration profiles, with C_max_ values in the fast group significantly higher than those in the medium and slow groups.

**Discussion:**

These findings demonstrate that injection rate directly influences both therapeutic and adverse effects through alterations in pharmacokinetic parameters, particularly C_max_. Setting a reasonable injection rate in animal experiments will show positive significance and help reduce related safety risks, especially in the application of anesthetics. In addition, strategically optimizing the injection rate during the development of innovative drugs is expected to improve the predictive validity of translational research.

## 1 Introduction

Innovative drug research and development (R&D) is widely recognized as a high-risk, high-reward endeavor that demands substantial time and financial investment. Typically, the development process encompasses several key phases: the exploratory phase, pharmacological research phase, preclinical biological research phase, clinical research phase, and postmarketing phase (1). The primary objective of preclinical research is to conduct preliminary evaluations and experimental studies on the drug, with animal experimentation serving as a central component. Animal models are essential tools for simulating human diseases and play a critical role in assessing drug safety and efficacy (2).

In animal experiments, optimizing the injection rate is of particular importance. The rate of drug administration can significantly influence both efficacy and toxicity by affecting peak concentration (C_max_), time to peak concentration (T_max_), and overall drug distribution (3). According to the study by Jin et al., administering propofol at a faster rate resulted in a shorter onset time of anesthesia, while slower administration was associated with reduced respiratory and circulatory depression (4). Additionally, rapid injection of propofol has been shown to lower the required dosage and minimize drug accumulation (5).

In certain drug development contexts, injection rate must be carefully controlled. Anesthetics with ultra-short onset times often exhibit dose-dependent adverse effects, such as hemodynamic and respiratory depression, particularly during rapid pharmacological activation (3, 6). Therefore, in preclinical studies involving novel anesthetic agents, it is crucial to rigorously characterize the relationship between injection rate, pharmacodynamics (PD), and pharmacokinetics (PK) in order to optimize both therapeutic efficacy and safety margins (7). This study selected propofol, etomidate and sufentanil based on their wide applicability and representativeness in clinical anesthesia practice (8, 9). Propofol is a commonly used intravenous sedative-hypnotic drug, which takes effect rapidly and has a smooth recovery (10). Etomidate has a relatively small impact on hemodynamics and is suitable for patients with unstable cardiovascular function (11). Sufentanil is a potent opioid analgesic, often used for intraoperative analgesia and anesthesia maintenance (12). All three are administered intravenously and take effect quickly, meeting the clinical requirements for intraoperative anesthesia and analgesia.

Although Jin et al. have clarified the impact of the injection rate of propofol in humans, the systematic effects of the injection rate on PD and PK in animal models, especially in the context of anesthesia, remain to be fully investigated (4). Given that animal anesthesia is a core component of numerous basic experiments and preclinical studies of new drugs, filling this research gap is of critical importance. The mechanisms by which injection rate influences PK parameters and whether PD responses demonstrate rate-dependent correlations remain to be fully elucidated. In this study, we systematically evaluated the effects of varying intravenous injection rates on both PK and PD profiles in animal models using bolus administrations of propofol, etomidate, and sufentanil. The results suggest that the injection rate is an important factor influencing the PK/PD behavior of these three drugs in rats. This approach can effectively reduce false positive or false negative results caused by improper injection rates in animal experiments, which is particularly important in critical experiments such as animal anesthesia. This not only enhances the overall success rate of drug development, but its research conclusions also provide practical insights for guiding clinical translation, especially offering direct evidence for optimizing the administration regimens of drugs (including anesthetics).

## 2 Materials and methods

### 2.1 Materials

Propofol injection was purchased from Sichuan Guorui, Co., Ltd. (Leshan, China). Etomidate injectable emulsion was bought from Nhwa Pharma, Co., Ltd. (Xuzhou, China). Sufentanil citrate injection was bought from Sinopharm Sichuan Medicines Group Co., Ltd. (Chengdu, China).

### 2.2 Animals and ethics

SPF-grade male Sprague–Dawley rats weighing 230–250 g were used in this study. All rats were bought from Chengdu Dossy Experimental Animals Co., Ltd. (Chengdu, China). Before each experiment, all rats adaptively fed for 1 week at a constant temperature under SPF conditions with food and water given ad libitum. All animal experiments were approved by the Animal Ethics Committee of West China Hospital, Sichuan University, Chengdu, China (approval no. 20240923011) and adhered strictly to the NIH Guide for the Care and Use of Laboratory Animals.

### 2.3 Study design

This study consisted of three main components: pharmacodynamics experiments, respiratory and hemodynamic monitoring experiments, and pharmacokinetics experiments. PK and PD evaluations were performed in distinct rat cohorts. Despite being conducted independently, both studies shared a common experimental design: identical drug doses were administered across all PK and PD experiments, with animals strictly stratified according to the same intravenous injection rates. Thereby ensuring full consistency in key dosing parameters between the two experimental sets.

In the pharmacodynamics experiments, all three drugs were administered to rats via manual intravenous injection through the tail vein. To minimize inter-operator variability and ensure consistent drug administration, all injections were performed by a single experienced investigator who had undergone extensive training to maintain a stable and reproducible injection rate. A highly visible digital timer was employed to provide real-time feedback, ensuring that each injection was completed within the predefined time window, thereby ensuring accurate and uniform drug delivery. The rats that received each drug were randomly divided into three groups (*n* = 6), with different injection rates: 0.06 mL/s (fast group), 0.02 mL/s (medium group), and 0.01 mL/s (slow group) (13, 14) (Table 1). The sample size of *n* = 6 per group was determined based on the exploratory objective of this study. This group size allows for the assessment of trends and biologically significant differences in pharmacological responses between injection rates, while rigorously adhering to the Reduction principle of the 3R guidelines by minimizing animal use. The total dose for each drug was set at twice the median effective dose (2 × ED_50_), which was determined based on prior animal studies using the up-and-down method (15), with an injection rate of 0.02 mL/s. The ED_50_ was determined based on predefined efficacy criteria: loss of righting reflex (LORR) in the forelimbs for propofol and etomidate, and loss of tail withdrawal reflex (LOTWR) for sufentanil (16, 17).

**Table 1:** Drug administration parameters of the three anesthetics.

Drug	ED_50_ (95% CI)	Injection dose (2 × ED_50_)	Injection volume	Route of administration	Injection rate
Propofol	6.20 mg/kg	12.40 mg/kg	0.60 mL	IV bolus*	0.06 mL/s (fast group)
Etomidate	0.92 mg/kg	1.84 mg/kg	0.60 mL	IV bolus*	0.02 mL/s (medium group)
Sufentanil	0.80 μg/kg	1.60 μg/kg	0.60 mL	IV bolus*	0.01 mL/s (slow group)

*IV, intravenous injection.

In the respiratory and hemodynamic monitoring experiments, each drug was administered to rats at a dose of 2 × ED_50_ via tail vein injection. The rats were randomly divided into three groups (*n* = 6), corresponding to the same injection rate: 0.06 mL/s (fast group), 0.02 mL/s (medium group), and 0.01 mL/s (slow group).

In the pharmacokinetics experiments, each drug was administered intravenously at a dose of 2 × ED_50_ through the tail vein. Rats were randomly assigned to three groups (*n* = 6), differentiated by injection rate: 0.06 mL/s (fast group), 0.02 mL/s (medium group), and 0.01 mL/s (slow group).

### 2.4 Pharmacodynamics experiments

#### 2.4.1 Sedation and myoclonus monitoring

For propofol and etomidate, sedation onset time, sedation maintenance time, sedation scores, incidence of myoclonus, and duration of myoclonus were recorded across the three experimental groups following drug administration.

A standardized scoring system was used to assess sedation levels, defined as follows: 0: Normal limb muscle tone with preserved spontaneous activity and alert responsiveness; 1: Pronounced thigmotactic behavior (rats prefer to remain near the periphery of the cage); 2: Impaired hindlimb coordination during backward movement (retropulsion ataxia); 3: Inability to maintain forelimb rearing beyond 60°, with evident gait ataxia; 4: Prone posture with complete loss of upright stance, requiring abdominal support for locomotion; 5: LORR, defined as the inability to correct body position. A sedation score of ≥2 was considered indicative of effective sedation and was used as the threshold for pharmacodynamic analysis (18).

Myoclonus was defined as sudden, involuntary muscular contractions or twitches, typically presenting as episodic movements involving the back, shoulders, hindlimbs, and toes. In more severe cases, these contractions may escalate to generalized tremors affecting all limbs and the entire body (19).

#### 2.4.2 Analgesia monitoring

For sufentanil, the three experimental groups were monitored for analgesic onset time and duration of analgesic maintenance following drug administration.

A serrated metal clamp was applied to the proximal tail region (1–2 cm from the base) of each rat for a 10-s duration. A positive nociceptive response was defined as the occurrence of at least one of the following: audible vocalization, vigorous escape attempts, or an orienting response directed toward the clamp. Antinociceptive efficacy was considered achieved when no behavioral responses were observed during the entire stimulation period (20).

### 2.5 Respiratory and hemodynamic monitoring

In each rat, percutaneous catheterization of the tail artery was performed under aseptic conditions using a 22-gauge intravenous catheter (Terumo, Tokyo, Japan). Continuous hemodynamic monitoring was conducted via this arterial line. Baseline mean arterial pressure (MAP) and respiratory rate (RR) were recorded during a 10-min baseline period prior to drug administration, and post-drug changes were monitored for 30 min using the BL-420F Bio-signal Acquisition System (TECHMAN, Chengdu, China).

### 2.6 Pharmacokinetics experiments

Under aseptic conditions, peripheral venous access was established in each rat through percutaneous puncture using a 24-gauge intravenous catheter (Terumo, Tokyo, Japan). Serial blood samples (300 μL each) were collected at predefined time points: pre-dose baseline; and post-dose at 1, 5, 10, 20, 30, and 60 min following injection.

#### 2.6.1 High-performance liquid chromatography (HPLC)/high performance liquid chromatography-mass spectrometry (HPLC-MS) analysis of biological samples

The plasma concentrations of propofol were quantified using HPLC, whereas etomidate and sufentanil were analyzed by HPLC-MS/MS.

For sample preparation, 50 μL of plasma was mixed with 150 μL of acetonitrile containing the corresponding internal standard (IS) to achieve protein precipitation. Following vortex mixing, samples were centrifuged at 20000 rpm and 4 °C for 10 min. The supernatant was then collected and used directly for injection and analysis.

Chromatographic and mass spectrometric parameters for each analyte are detailed in Table 2. All compounds were qualitatively confirmed by matching retention times to authentic reference standards and were quantified using calibration curves generated from known standard concentrations (Table 2).

**Table 2:** Chromatographic and mass spectrometric conditions for the analysis of propofol, etomidate, and sufentanil.

Parameter	Propofol (HPLC-FLD)	Etomidate (HPLC-MS/MS)	Sufentanil (HPLC-MS/MS)
System	Agilent 1,200 HPLC	Agilent 1,260 HPLC – G6460 MS	Agilent 1,260 HPLC – G6460 MS
Column	Swell Chromplus C18 (4.6 × 150 mm, 5 μm)	Agilent Extend-C18 (3.0 × 100 mm, 3.5 μm)	Agilent Extend-C18 (3.0 × 100 mm, 3.5 μm)
Column temp.	30 °C	30 °C	30 °C
Mobile phase	0.1% TFA aq.: Acetonitrile (32:68, v/v)	(A) 0.1% Formic acid aq.; (B) Acetonitrile	(A) 0.1% Formic acid aq.; (B) Acetonitrile
Gradient	Isocratic	16% B (0–2 min) → 90% B (4–7 min)	35% B (0–2 min) → 90% B (3–6 min)
Flow rate	1.0 mL/min	300 μL/min	300 μL/min
Detection	Fluorescence (FLD) λ_ex_ 276 nm/λ_em_ 310 nm	Mass spectrometry (MS/MS)	Mass spectrometry (MS/MS)
Ionization mode	Not applicable	Electrospray ionization, positive	Electrospray ionization, positive
Monitoring mode	Not applicable	Multiple reaction monitoring	Multiple reaction monitoring
Quantification ion pair (m/z)	Not applicable	245 → 141	387.2 → 111.1
IS ion pair (m/z)	Not applicable	259 → 155.1	259 → 155.1
Internal standard (IS)	Thymol (500 ng/mL in ACN)	Etomidate impurity C (10 ng/mL in ACN)	Etomidate impurity C (10 ng/mL in ACN)

### 2.7 Statistical analysis

Data analyses were performed using GraphPad Prism 10.4.1 (GraphPad Software Inc., San Diego, CA, USA). Results are expressed as mean ± SD. Statistical significance was defined as *p* < 0.05. Continuous data were assessed for normality using the Shapiro–Wilk test. Continuous data with normality were compared using the ordinary one-way ANOVA. Two-by-two comparisons were made between groups using the Tukey’s multiple comparisons test. Comparisons of continuous data that do not have normality were tested using Kruskal-Wallis test. Two-by-two comparisons were made between groups using the Dunn’s multiple comparisons test. Two independent categorical variables with normality were compared using the two-way ANOVA. For two independent groups of continuous data with unequal variances, Welch’s t-test was applied.

## 3 Results

### 3.1 Faster injection rate leads to shorter onset time and longer maintenance duration

**Figure 1:**
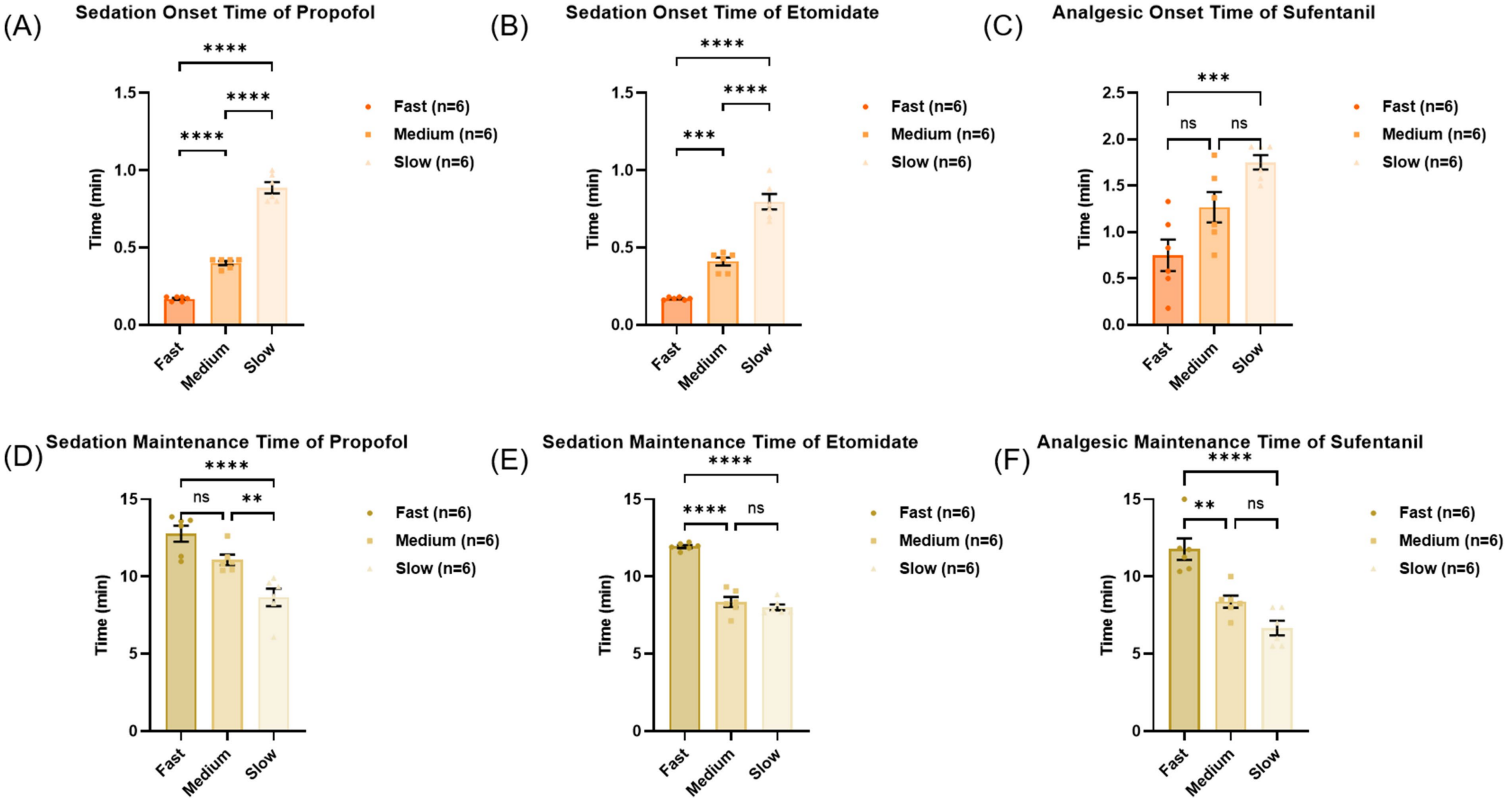
Onset and maintenance time in three groups of propofol, etomidate and sufentanil. **(A)** Sedation onset time in three groups of propofol, *p* < 0.0001. **(B)** Sedation onset time in three groups of etomidate, *p* < 0.0001. **(C)** Analgesic onset time in three groups of sufentanil, *p* < 0.0001. **(D)** Sedation maintenance time in three groups of propofol, *p* < 0.001. **(E)** Sedation maintenance time in three groups of etomidate, *p* < 0.0001. **(F)** Analgesic maintenance time in three groups of sufentanil, *p* < 0.0001. Results are expressed as mean ± SD. Statistical significance was determined by one-way ANOVA. ns: no significance, *p < 0.05, **p < 0.01, ***p < 0.001 and ****p < 0.0001; (*n* = 6).

The injection rate significantly influences both the onset and maintenance duration of drug effects. For propofol and etomidate (Figures 1A,B), the onset time of fast administration was significantly shorter than that of medium-speed administration, and the onset time of medium-speed administration was significantly shorter than that of slow administration. However, for sufentanil (Figure 1C), the statistical results showed that the onset time of fast administration was significantly shorter than that of slow administration, but no statistically significant differences were observed between fast and medium-speed administration, or between medium-speed and slow administration. With regard to sedation maintenance, the duration was significantly longer in the fast group than in the slow group for propofol, and in the medium group than in the slow group (Figure 1D). However, no significant difference was observed between the fast and medium groups. For etomidate, sedation maintenance time was significantly longer in the fast group compared to both the medium and slow groups, with no significant difference between the latter two (Figure 1E). For sufentanil, analgesia maintenance time was significantly prolonged in the fast group compared to the medium and slow groups, while no significant difference was found between the medium and slow groups (Figure 1F). Overall, a faster injection rate resulted in a shorter onset latency and a longer duration of effect maintenance.

### 3.2 Faster injection rate exacerbates respiratory depression

**Figure 2:**
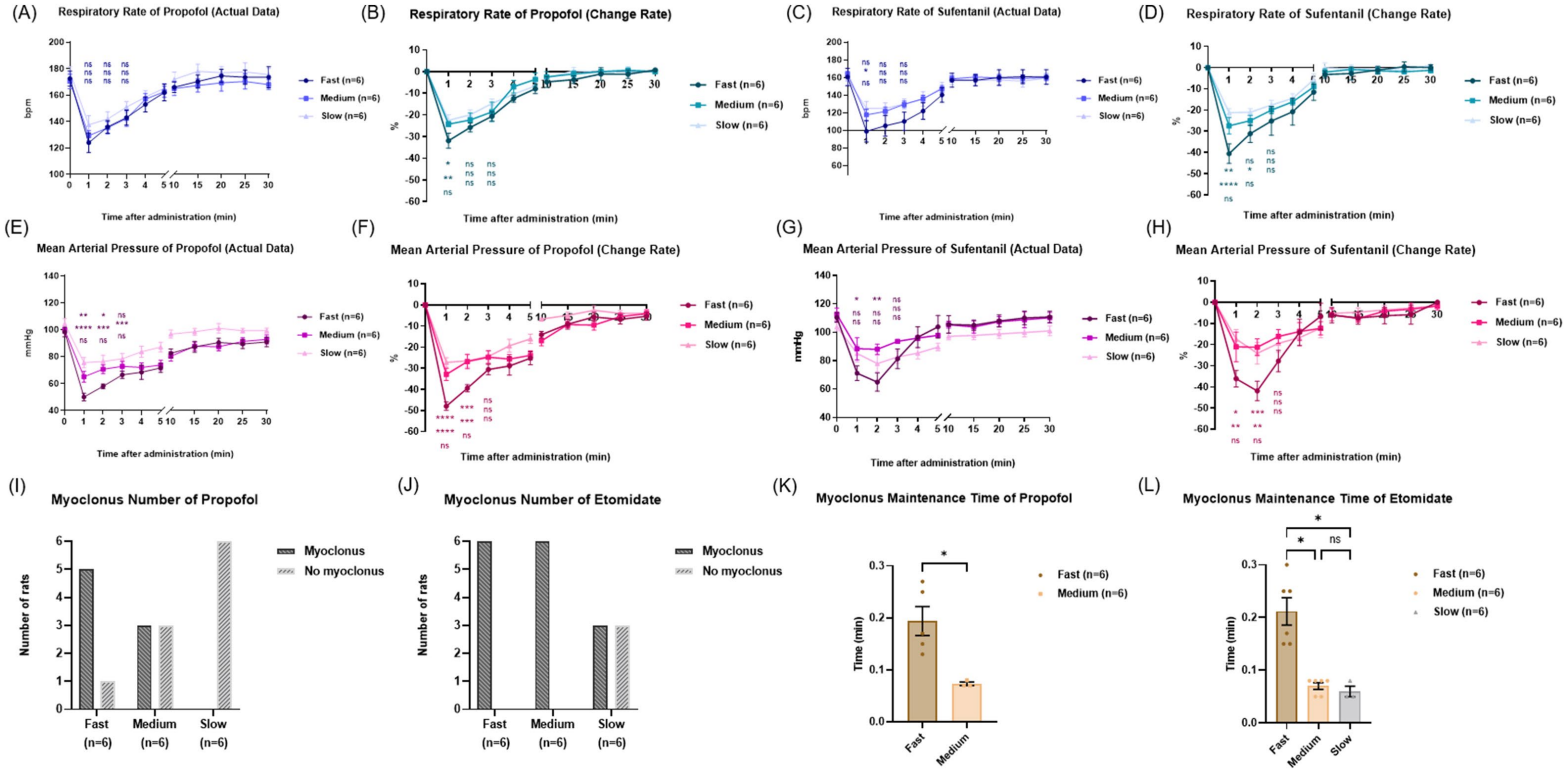
Side effects in the three groups for propofol, etomidate and sufentanil. **(A)** Respiratory actual rate of propofol. **(B)** Respiratory actual rate of sufentanil. **(C)** Respiratory change rate of propofol. **(D)** Respiratory change rate of sufentanil. Results are expressed as mean ± SEM; statistical significance was determined by two-way ANOVA. ns: no significance. **(E)** Circulatory actual rate of propofol. **(F)** Circulatory actual rate of sufentanil. **(G)** Circulatory change rate of propofol. **(H)** Circulatory change rate of sufentanil. **(I)** Myoclonus episode number of propofol. **(J)** Myoclonus episode numbers of etomidate. **(K)** Myoclonus maintenance time of propofol, *p* < 0.05. **(L)** Myoclonus maintenance time of etomidate, *p* < 0.001. Results are expressed as mean ± SD. ns, no significance; *p < 0.05, **p < 0.01, ***p < 0.001 and ****p < 0.0001. **(A–H)** Statistical analysis of the first 3 min in order from top to bottom: Fast group vs. Medium group, Fast group vs. Slow group, Medium group vs. Slow group; (*n* = 6).

Respiratory depression is a prominent adverse effect associated with the administration of propofol and sufentanil (21, 22). In the three groups of experiments with the two drugs, a rapid decrease in RR was observed early after administration (1–3 min) (Figures 2A,B). For propofol, the fast group experienced the greatest decrease in RR early after administration, reaching 35%, which was significantly higher than the medium group (24%) and the slow group (22%) (Figure 2C). Similarly, for sufentanil, the fast group had the greatest decrease in RR early after administration, reaching 40%, while the medium group was 28% and the slow group was 21% (Figure 2D). These results indicate that the faster the injection rate of propofol and sufentanil, the more severe the respiratory depression, while a slower injection rate is associated with relatively mild respiratory adverse reactions.

### 3.3 Faster injection rate exacerbates circulatory depression

Circulatory depression is another major adverse effect associated with the administration of propofol and sufentanil (8, 23). Shortly after the injection of propofol (1–3 min), the MAP rapidly decreased in all three infusion rate groups (Figure 2E). For sufentanil, the fast group also experienced a sharp drop in MAP in the early stage after injection (1–3 min), while the changes in the medium group and the slow group were relatively small (Figure 2F). In the fast propofol group, the maximum decrease in MAP in the early stage reached 49%, which was higher than that in the medium group (33%) and the slow group (28%) (Figure 2G). Similarly, for sufentanil, the maximum decrease in MAP in the early stage was 37% in the fast group, 21% in the medium group, and 18% in the slow group (Figure 2H). These findings suggest that the faster the injection rate, the more pronounced the circulatory suppression, manifested as a greater decrease in blood pressure, while a slower injection rate is associated with milder hemodynamic disturbances.

### 3.4 Elevated episode frequency and prolonged duration of myoclonus following faster injection rate

Propofol and etomidate are associated with the adverse effect of myoclonus within 1–3 min of administration (24, 25). The incidence of myoclonus was 5/6 in the fast group, 3/6 in the medium group, and 0 in the slow group for propofol (Figure 2I). Myoclonus was observed in all 6 of the fast and medium groups for etomidate, and in 3/6 of the slow group (Figure 2J). The duration of myoclonus maintenance was greater in the fast groups than in both the medium and slow groups for propofol and etomidate (Figures 2K,L). Faster injection rate led to an increase in the number of propofol myoclonus occurrences and prolonged propofol and etomidate myoclonus maintenance time.

### 3.5 Injection rate alters certain pharmacokinetic parameters

**Figure 3:**
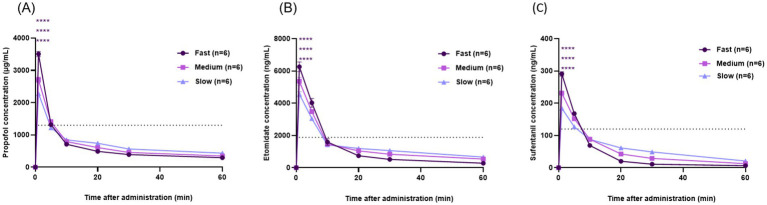
Time-concentration curve in three groups of propofol, etomidate and sufentanil. **(A)** Time-concentration curve of propofol. **(B)** Time-concentration curve of etomidate. **(C)** Time-concentration curve of sufentanil. Results are expressed as mean ± SD. ns, no significance; *p < 0.05, **p < 0.01, ***p < 0.001 and ****p < 0.0001. Statistical analysis of the first 3 min in order from top to bottom: Fast group vs. Medium group, Fast group vs. Slow group, Medium group vs. Slow group; (*n* = 6).

The blood concentrations of propofol, etomidate, and sufentanil all reached their peak at 1 min following administration, with the C_max_ in the fast injection group being higher than that in the medium group, which in turn was higher than that in the slow group. The horizontal dashed lines in Figures 3A–C represent the plasma concentrations corresponding to 2 × ED_50_ of LORR/LOTWR, which are derived from our own PD data. Although the correlation between C_max_ and adverse effects has been clearly confirmed, the time during which the blood drug concentration is above the effective threshold does not fully support the explanation that the prolonged duration of action is driven by C_max_. This is because this study, as an initial PK exploration, did not directly measure the drug concentration at the site of action (such as the central nervous system). However, the “effect compartment model” in the classic PK-PD theory provides a reasonable explanation for this, which clarifies the time delay between plasma concentration and drug effect, which is particularly important for centrally acting drugs (26) (Figure 3; Table 3).

**Table 3:** PK parameters for the three anesthetics groups.

Parameter	Propofol			Etomidate			Sufentanil		
	Fast group	Medium group	Slow group	Fast group	Medium group	Slow group	Fast group	Medium group	Slow group
C_max_ *(ng/mL)	3,508 ± 186	2,706 ± 200	2,282 ± 152	6,262 ± 740	5,358 ± 589	4,561 ± 379	291 ± 18	231 ± 13	184 ± 10
T_max_ (min)	1	1	1	1	1	1	1	1	1
AUC *(min·ng/mL)	33,896 ± 1,142	32,649 ± 682	32,331 ± 1,434	97,908 ± 3,436	94,478 ± 3,390	98,353 ± 3,534	1,550 ± 102	1,682 ± 111	1,478 ± 124

*Mean ± SD.

## 4 Discussion

This systematic study elucidated the pharmacodynamic and pharmacokinetic effects of three clinically relevant anesthetics (propofol, etomidate, and sufentanil) administered intravenously on rats. Our data demonstrate that a fast injection rate (0.06 mL/s) significantly alters both therapeutic indices and safety profiles, as evidenced by a reduced onset latency and an increased severity of side effects compared to a slow injection rate (0.01 mL/s). The observed hemodynamic perturbations, including transient hypotension and respiratory depression, are consistent with pharmacokinetic findings showing dose-rate-dependent elevations in C_max_.

In the context of this study, the PD endpoints of sedation (LORR) and analgesia (LOTWR) require rapid onset and are critically dependent on achieving a high initial drug concentration at the effect site. A higher C_max_ facilitates more rapid and extensive distribution to the central nervous system, enabling the effect-site concentration to surpass the pharmacological threshold decisively and remain above it for an extended duration under the influence of the initial plasma peak (27, 28). This mechanism accounts for the observed correlation between elevated C_max_ and prolonged duration of action. Although the plasma concentration-time profiles may appear discordant with the time course of pharmacological effects at first glance, this discrepancy is well explained by the established “effect compartment model,” which describes the hysteresis between plasma concentrations and clinical effects, particularly relevant for centrally acting agents (26, 29). *In vivo* real-time measurement of effect compartment concentrations presents significant technical challenges (29). Beyond C_max_, future studies should systematically evaluate other key PK parameters that may influence the maintenance phase. The equilibrium rate constant (ke0), elimination half-life (t_1/2_), and mean residence time (MRT) each offer potential to more accurately reflect total pharmacologically relevant exposure, particularly in the presence of kinetic delays between compartments (30).

Previous clinical studies have investigated the appropriate concentration or dosage of anesthetics to be administered during anesthesia. However, few reports have specifically explored the optimal injection rate of anesthetics in animal experiments (31, 32). Sepulveda et al. compared changes in plasma drug concentrations in patients receiving propofol infusions at different rates and administered a loading dose over (36 ± 9) seconds (31). Based on this, the injection duration in the medium group was set at 30 s in this study. Struys et al. used electroencephalography to examine the influence of intravenous administration rate on the equilibration of blood concentration and administered a loading dose within 10 s (14). Blum et al. compared the effects of injecting anesthetics over durations of 5, 120, and 240 s on the depth of sedation (33). Consequently, in our study, the injection time was set at 10 s for the fast group (0.06 mL/s) and 60 s for the slow group (0.01 mL/s). The terms “fast group” and “slow group” are used comparatively to explore suitable drug administration rates in animal experiments. In actual experimental settings, the injection rate must be adjusted according to various factors, including species, sex, and age.

In addition to the injection rate, factors such as needle specifications (size) and injection angle also significantly affect the flow and distribution characteristics of the drug solution within the tissue. These parameters are directly related to the local drug concentration, diffusion range, and tissue retention time, thereby influencing the absorption rate and bioavailability of the drug. Therefore, they are of great significance in PD and PK research. Especially in the process of new drug development, a systematic assessment of injection technique-related variables plays a crucial role in understanding the intrinsic mechanism of drug delivery in preclinical animal models and clinical human trials (34, 35).

Several methodological limitations in the present study warrant careful consideration. First, the use of a fixed dosing regimen at 2 × ED_50_, although effective in standardizing pharmacodynamic responses across subjects, may have obscured more subtle, injection rate-dependent pharmacological effects due to potential receptor saturation at higher concentrations (36, 37). Future investigations would benefit from incorporating dose-titration designs (e.g., spanning 0.5–3 × ED_50_) to systematically elucidate the interplay between injection rate, plasma concentration profiles, and resultant pharmacodynamic outcomes. Second, the single-bolus administration paradigm employed here does not fully replicate clinical anesthesia practice, in which continuous infusion protocols are commonly used. Subsequent studies should implement pump-controlled infusion systems, particularly those guided by real-time pharmacokinetic modeling such as target-controlled infusion, to more accurately simulate clinical drug delivery and enable precise control of plasma and effect-site concentrations. Third, technical challenges associated with ultra-early-phase plasma sampling (*t* < 1 min after administration) restrict the precise analysis of the initial drug distribution kinetics, thereby hindering the complete PK-PD modeling (as the onset time of most drug effects is within 1 min). Advancements in rapid-sampling methodologies will be necessary to accurately capture the earliest phases of drug disposition. For instance, invasive femoral artery blood sampling under isoflurane anesthesia can collect blood more frequently (e.g., every 30 s), thereby more accurately determining the C_max_ value of the drug and establishing the connection between PD and PK within the first minute after administration. In addition, only male animals were used in this study. Future research should include female animals to systematically investigate potential gender-specific differences.

For well-established therapeutics such as these, modeling and simulation approaches offer a viable alternative to animal studies, thereby better aligning with the 3R principles. However, as this is the first study to directly examine the influence of injection rate on the PK/PD behavior of these agents, *in vivo* experiments were essential to establish a robust and physiologically relevant evidence base. Moreover, such experimental models may uncover emergent responses that are difficult to anticipate through simulation alone. The data generated from this study provide a solid platform for future mechanism-based PK/PD modeling and simulation efforts, enabling a deeper understanding of administration rate effects and supporting the development of more predictive, reproducible, and ethically sound research frameworks in line.

Preclinical research, especially animal experiments, is a key component of innovative drug development. By generating robust and reliable data, these studies provide a foundational basis for subsequent human investigations. Within this framework, the standardization of administration protocols, particularly the injection rate in animal models, is of considerable methodological importance. Controlling the injection rate reduces pharmacokinetic variability and decreases the risk of false-negative results caused by inadequate drug exposure or rapid clearance. Optimizing such parameters enhances the reproducibility, predictive validity, and regulatory acceptability of preclinical findings, thereby facilitating a smoother transition to clinical phases and accelerating overall drug development timelines. Therefore, it is strongly recommended that preliminary dose-ranging and injection optimization studies be conducted prior to pivotal animal trials to identify an injection rate that maximizes the therapeutic index while minimizing adverse effects. In addition, the methodology established in this study can serve as a reference for other pharmacological and toxicological research based on animal models, especially in the field of animal anesthesia, standardizing drug administration techniques, reducing experimental variability, and enhancing the reproducibility of results. To a certain extent, it promotes the standardization and scientific nature of preclinical animal medical research.

## 5 Conclusion

This study confirms that injection rate has a definitive impact on animal experiments. The injection rate of a bolus dose of an intravenous anesthetic influence both PD and PK outcomes. Rapid administration of anesthetics prolongs the duration of action, whereas slower injection reduces the likelihood of adverse effects. Faster injection may lead to higher C_max_. In animal experimentation, the influence of injection rate should be carefully considered. Selecting an appropriate injection rate can minimize false-positive and false-negative results, thereby enhancing the reliability and efficiency of innovative drug development. The findings and conclusions of this study are based on an analysis of the tested anesthetic/analgesic drugs (propofol, etomidate and remifentanil).

## Data Availability

The raw data supporting the conclusions of this article will be made available by the authors, without undue reservation.
